# Structural Basis of Bifunctional CTP/dCTP Synthase

**DOI:** 10.1016/j.jmb.2024.168750

**Published:** 2024-08-20

**Authors:** Chen-Jun Guo, Zherong Zhang, Jia-Li Lu, Jiale Zhong, Yu-Fen Wu, Shu-Ying Guo, Ji-Long Liu

**Affiliations:** 1 School of Life Science and Technology, ShanghaiTech University, Shanghai 201210, China; 2 Department of Psychological and Brain Sciences, Dartmouth College, Hanover, NH 03755, USA; 3 Department of Physiology, Anatomy and Genetics, University of Oxford, Oxford OX1 3PT, United Kingdom

**Keywords:** CTP synthase, CTP/dCTP synthase, cryo-electron microscopy, cytoophidium, filamentation

## Abstract

The final step in the de novo synthesis of cytidine 5′-triphosphate (CTP) is catalyzed by CTP synthase (CTPS), which can form cytoophidia in all three domains of life. Recently, we have discovered that CTPS binds to ribonucleotides (NTPs) to form filaments, and have successfully resolved the structures of *Drosophila melanogaster* CTPS bound with NTPs. Previous biochemical studies have shown that CTPS can bind to deoxyribonucleotides (dNTPs) to produce 2′ -deoxycytidine-5′ -triphosphate (dCTP). However, the structural basis of CTPS binding to dNTPs is still unclear. In this study, we find that *Drosophila* CTPS can also form filaments with dNTPs. Using cryo-electron microscopy, we are able to resolve the structure of *Drosophila melanogaster* CTPS bound to dNTPs with a resolution of up to 2.7 Å. By combining these structural findings with biochemical analysis, we compare the binding and reaction characteristics of NTPs and dNTPs with CTPS. Our results indicate that the same enzyme can act bifunctionally as CTP/dCTP synthase *in vitro*, and provide a structural basis for these activities.

## Introduction

The final step in the de novo synthesis of cytidine 5′-triphosphate (CTP) is catalyzed by CTP synthase (CTPS). CTPS plays a critical role in the synthesis of nucleic acids and phospholipids in living systems.^1–4^ Due to its upregulation in various types of cancer, CTPS is considered an attractive target for combating cancer^5–12^ and immunosuppression,^13,14^ and controlling infections caused by protozoa,^15^ viruses,^16^ and *Mycobacterium tuberculosis*.^17–19^

CTPS is composed of two distinct domains: the N-terminal ammonia ligase (AL) domain and the C-terminal glutamine amide transferase (GAT) domain. In the AL domain, UTP is phosphorylated by hydrolyzing ATP, which activates UTP. Then, in the presence of ammonia, the intermediate 4Pi-UTP is converted into CTP. The ammonia necessary for this reaction is produced through the hydrolysis of glutamine in the GAT domain and then transferred to the AL domain through the ammonia tunnel.^3,20–26^ Throughout the entire process, the allosteric regulator GTP plays a crucial role in coordinating the activity of the two domains and is essential for constructing the ammonia tunnel.^20,22–24,26–33^

The regulation of CTPS by its ligands has been studied for many decades, with a focus on feedback inhibition by NADH and CTP, as well as allosteric regulation by GTP.^8,21,22,26,30,34–37^ It has been found that GTP has a unique impact on the regulation of CTPS. Specifically, GTP is necessary when utilizing glutamine as the source of ammonia.^20,23,24,26,30,32,33,38^ At lower concentrations, GTP enhances the catalytic activity of CTPS, but as the concentration increases, its stimulating effect weakens. It is important to note that exceeding the optimal level of GTP can actually have a negative impact on CTPS activity.

The binding sites of various ligands of CTPS were determined by means of X-ray crystallography or cryo-electron microscopy (cryo-EM). The binding pockets of ADP and CTP were first visualized by Endrizzi et al. in *Escherichia coli* CTPS, while Lynch et al. showed the binding pockets of ATP and UTP in human CTPS1.^14,33,39–44^ In addition, CTPS is also regulated by post-translational modifications such as phosphorylation, which has been validated in different species.^45–49^

Previous studies have shown that CTPS can form filamentous structures, known as ‘cytoophidia’, *in vivo*^50–52^. These structures are conserved in both prokaryotes and eukaryotes.^53–62^ *In vitro* studies have also demonstrated that CTPS proteins from various species have the ability to form metabolic filaments when binding with substrates and/or products.^13,14,41–43,61,63–65^ These CTPS filaments are considered to be the fundamental components of cytoophidia in vivo. Through a combination of structural studies and kinetic assays, researchers have discovered that the regulation of CTPS through fila-mentation is complex and diverse.^41,57^ For example, while *Escherichia coli* CTPS forms filaments and inhibits enzymatic activity under product-binding conditions, *Drosophila* CTPS filaments can form in the presence of either product or substrate.^41,57,66^

In 1999, Carman’s group discovered a potential method in which URA7 (also known as CTPS) from *Saccharomyces cerevisiae* could catalyze the biosynthesis of dCTP.^67^ This research suggested that CTPS could also function as a dCTP synthase (dCTPS). To avoid confusion, we will continue to use the term “CTPS” to refer to CTP/dCTP synthase. However, the structural basis of this phenomenon is still unclear.

Using cryo-EM, we have successfully determined the structure of *Drosophila* CTPS containing dNTPs and the inhibitor 6-diazo-L-noreleucine (DON). Through a combination of kinetic assays and other methods, we have found that: (1) dATP acts similarly to ATP as a substrate; (2) dUTP is a substrate with low reaction efficiency; (3) dGTP is a weaker allosteric regulator than GTP; and (4) dCTP is a strong inhibitor for *Drosophila* CTPS. Based on these results, we have proposed a diagram illustrating the bifunctionality of CTPS.

## Results

### Determination of the dynamic structure of CTPS under dNTP substrate conditions

**Figure 1:**
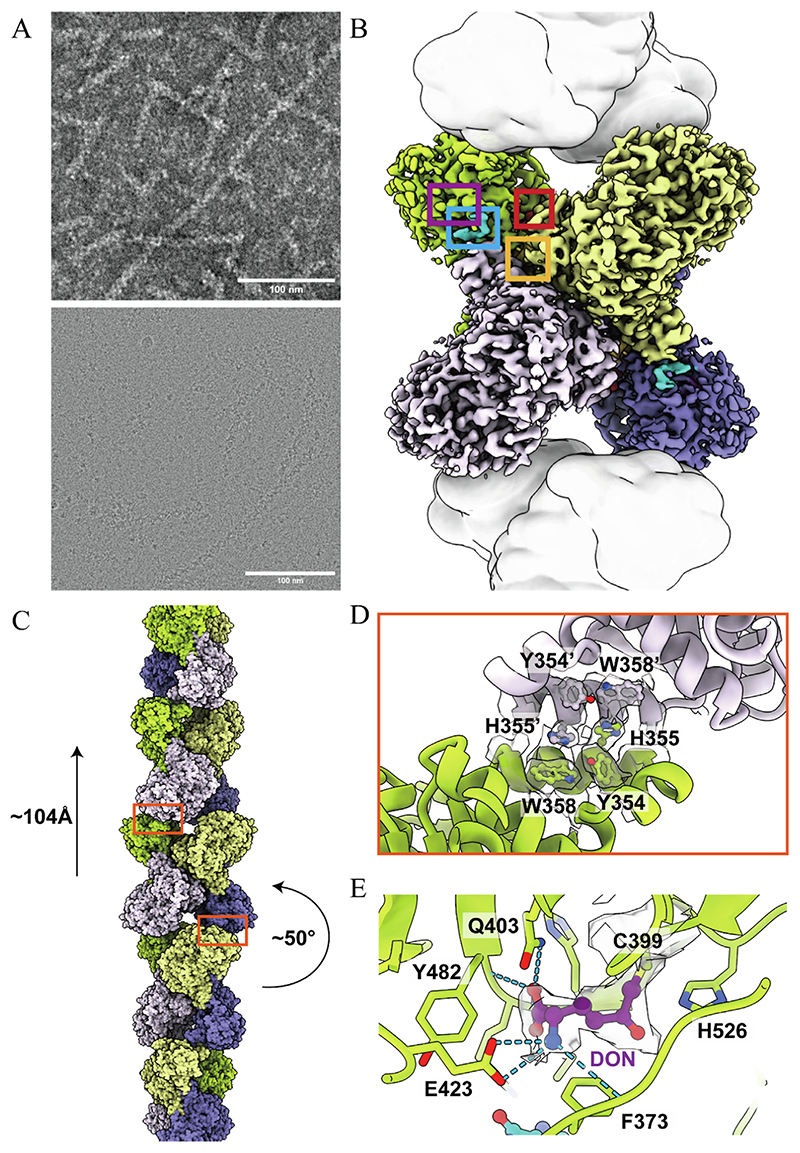
The overall structure of CTPS with dNTPs and DON. A, Negative staining electron microscopy images of 1.76 μM CTPS mixed with 2 mM dATP, 2 mM dUTP, 2 mM dGTP, 0.6 mM DON, and 8 mM MgCl_2_, as well as filtered images of cryo-EM sample under dNTP substrate conditions. Scale bars, 100 nm. B, The 2.7 Å resolution structure of a CTPS tetramer, with each protomer having a different color. Adjacent CTPS tetramers that form filaments with the tetramer in center are indicated by white surface. The binding sites for glutamine, UTP, ATP and GTP are indicated by purple, red, yellow and blue box, respectively. C, The overall structure model of CTPS filament and its helical parameters: rising by 104 Å, and twisting by 50°, as shown in the figure. D, CTPS filament interface model. E, Covalent binding between DON and C399. Hydrogen bonds are indicated by blue dashes. The map density of DON is displayed on a transparent surface.

Usually, the filamentation of *Drosophila* CTPS requires the presence of AL domain ligands (Supplementary Figure S1). Interestingly, using negative-staining EM, we observed that *Drosophila* CTPS mixed with dATP, dUTP and dGTP can form filaments. The same phenomenon was directly observed in cryo-EM samples (Figure 1A). This prompted us to investigate the role of dNTPs in CTPS catalysis and their respective structural basis.

Therefore, we used cryo-EM to determine and acquire the structure of CTPS from *Drosophila melanogaster* under dNTP substrate conditions with a resolution of up to 2.7 Å, which is referred to as dDON model from here on (Table S1, Supplementary Figure S2-4). In the dDON model, CTPS forms tetramers (Figure 1B) and then assembles into filaments (Figure 1C). The helical rise is 104 Å, and the twist is 50°. The CTPS tetramers form filaments at the tetramer-tetramer interface through 355H (Figure 1D).

The electron density of the covalent inhibitor DON was observed in the binding pocket of the GAT domain, which is related to the density of the catalytic amino acid C399 (Figure 1E). This acquired structure provides a structural basis for the formation of CTPS filaments stimulated by dNTPs.

### The role of dATP as a catalytic substrate for CTPS is similar to that of ATP

**Figure 2:**
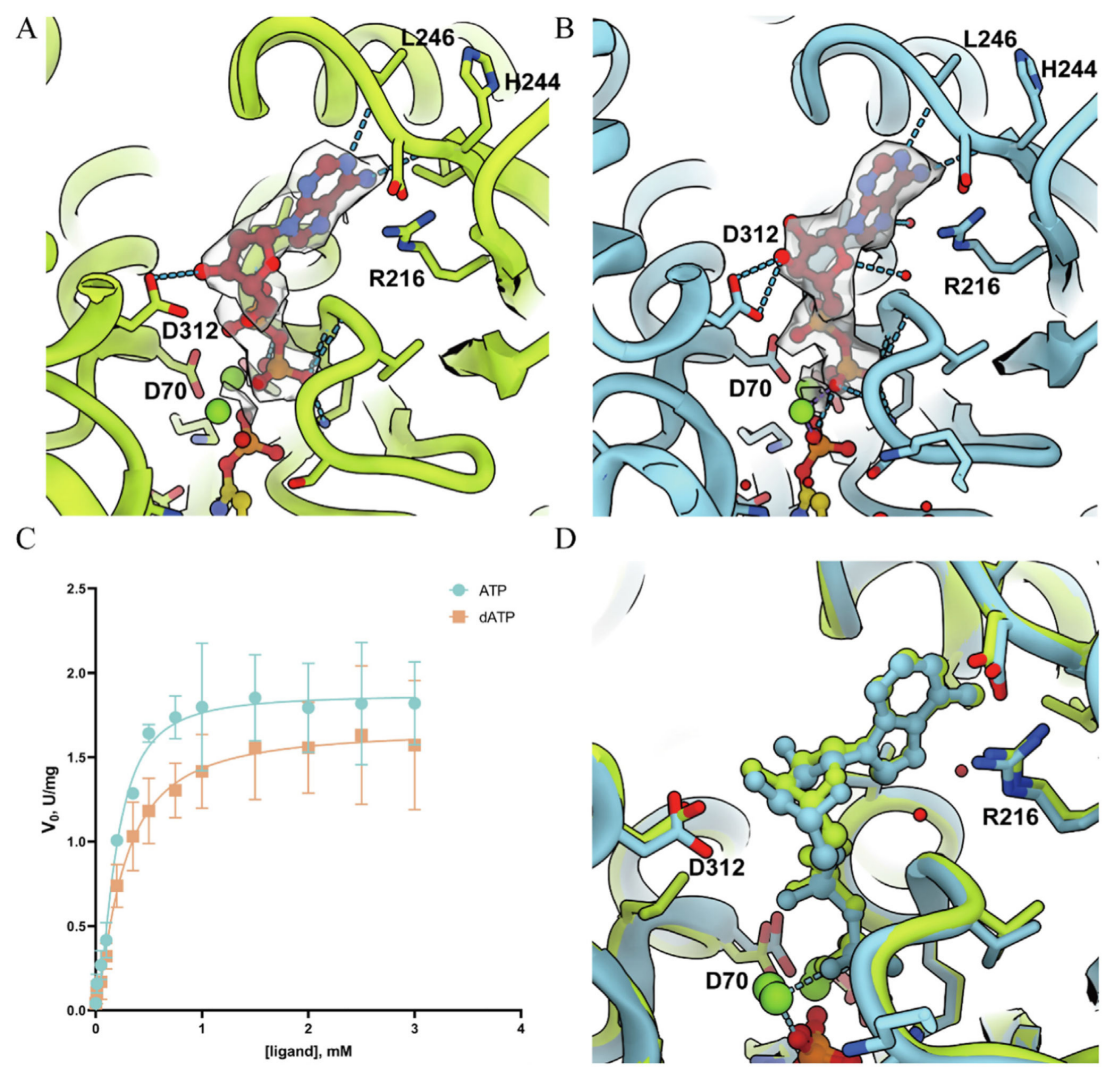
The structural and kinetic characteristics of CTPS in ATP and dATP binding modes. A, dATP binding site in CTPS under dDON state (in this study). Mapping counter level: 7σ. B, ATP binding site in CTPS under DON state (PDB 7DPT). Mapping counter level: 7 σ. C, Kinetic curves of CTPS in both ATP and dATP binding modes. The X-axis indicates ligand concentration. The Y-axis indicates the initial velocity of the enzymatic reaction. We chose the Hill equation for fitting because enzymes exhibit cooperativity, and their activity is more in line with the Hill Equation than other models. D, Structural comparison between ATP binding pocket in DON state (blue) and dATP binding pocket in dDON state (green).

Based on our high-resolution electron density map, we accurately identified the density of dATP in the pocket (Figure 2A). Consistent with the structure obtained in the presence of ATP, UTP, inhibitor DON, and GTP (referred to as DON state, PDB ID: 7DPT), dATP has been converted to dADP by CTPS (Figure 2B).

The binding recognition of dADP exhibits similar characteristics to ADP, where two phosphate groups are stabilized by electrostatic interactions between the N-terminus of helix 16–29 and the backbone of loop 12–15. The ribose portion forms hydrogen bonds with D312. The adenine moiety is stabilized through two hydrogen bonds with H244 and L246, as well as π-π interaction with R216 (Figure 2A, B). At the 7σ contour level, two maps of dDON and DON states show the density differences near the ribose portion, reflecting different binding ligands.

In terms of catalytic behavior, Endrizzi et al. previously demonstrated that dATP is equivalent to ATP in *E. coli* CTPS.^44^ We fitted the ATP-driven and dATP-driven kinetic curves using the Hill equation (Figure 2C). The Hill equation is as follows:

$$V_{0}=\frac{V_{max}*{\left[ATP\;or\;dATP\right]}^{n}}{{K_{A}}^{n}+{\left[ATP\;or\;dATP\right]}^{n}}$$

where $K_{d}={K_{A}}^{n}$

The *V_max_, n, K_A_*, and *K_d_* of ATP-driven activity are 1.87 ± 0.06 U/mg, 1.67 ± 0.25, 0.19 ± 0.02 mM, 0. 06 ± 0.03 mM, respectively. The *V_max_, n, K_A_*, and *K_d_* of dATP-driven activity are 1.67 ± 0.10 U/mg, 1.33 ± 0.26, 0.25 ± 0.04 mM, 0.16 ± 0.08 mM, respectively (Table S2).

The *V_max_* displayed by the ATP curve is usually higher than that of the dATP curve, which means that CTPS reacts faster to ATP than dATP. The lower K_d_ (measuring overall binding affinity) of ATP-driven activity indicates that CTPS prefers to bind to ATP than dATP. In terms of cooperativity, as measured by the Hill coefficient n, both ATP- and dATP-driven activities exhibit positive cooperativity, because their respective Hill coefficients are greater than 1.

To find the structural basis for kinetic observations, we compared the CTPS structure of dDON state with the DON state structure. They are aligned by the first 42 amino acids, which are the main components of the ligand binding pockets of the AL domain. Compared with the DON state, the overall conformation of the ATP binding pocket is similar, with a root-mean-square deviation (RMSD) of 0.373 Å for amino acids 1–42 (Figure 2D).

The ligand binding mode also exhibits a similar pattern, but compared to ribose, the position of deoxyribose has undergone a significant shift. Specifically, the deoxyribose C3′ atom has been displaced by 0.8 Å, located between the ribose C2′ and C3′ atoms. This shift can be attributed to the different structural flexibilities of deoxyribose and ribose, as well as their different hydrogen bonding interactions with amino acid D312. These structural evidences may be the basis for the differences in kinetic parameters between ATP driven enzyme activity and dATP driven enzyme activity.

In addition, no direct interaction between amino acids and ribose 2′OH was observed in the DON state model, indicating that the absence of 2′OH functional groups may not have a significant impact on ligand recognition.

### dUTP acts as a less efficient substrate for CTPS activity than UTP

**Figure 3:**
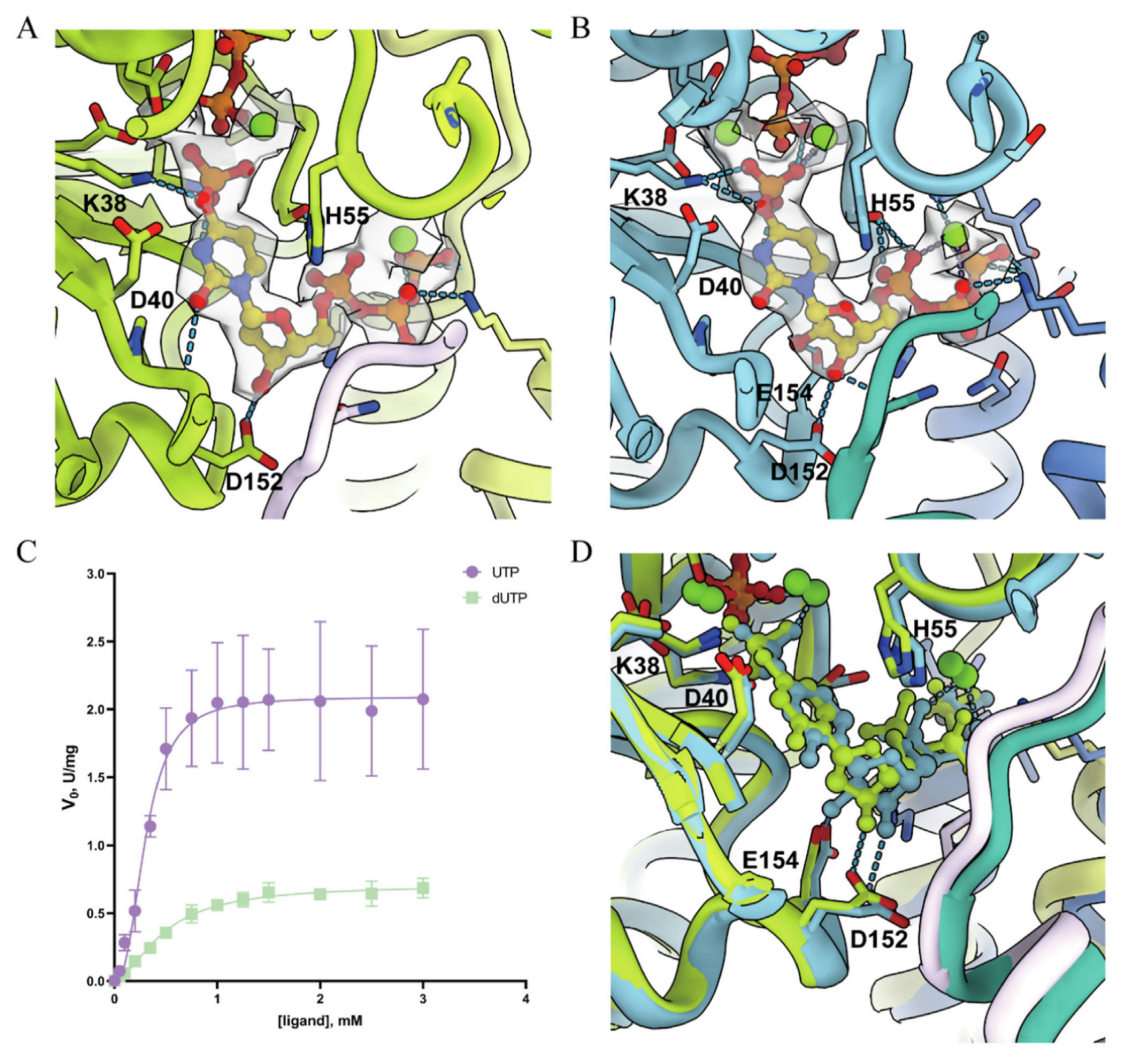
The structural and kinetic characteristics of CTPS in UTP and dUTP binding modes. A, dUTP binding site in CTPS under dDON state (in this study). Mapping counter level: 7σ. B, UTP binding site in CTPS under DON state (PDB 7DPT). Mapping counter level: 7σ. C, Kinetic curves of CTPS activity using UTP and dUTP as substrates. The X-axis indicates ligand concentration. The Y-axis indicates the initial velocity of the enzymatic reaction. We chose the Hill equation for fitting because enzymes exhibit cooperativity, and their activity is more in line with the Hill Equation than other models. D, Structural comparison between UTP binding pocket in DON state (blue) and dUTP binding pocket in dDON state (green).

We have also located and analyzed the position of dUTP in its binding pocket of CTPS (Figure 3A). Similar to the DON state, our electron density map shows that dUTP has been catalyzed by CTPS to 4Pi-dUTP and stabilized through various interactions (Figure 3B).

Then, we performed kinetic measurements of CTPS activities using UTP as substrate (UTP activity) and dUTP as substrate (dUTP activity) (Figure 3C). The curves showed significant differences in the binding affinity of CTPS to each substrate.

We fitted the activity of UTP and dUTP using the Hill equation. The equation is as follows:

$$V_{0}=\frac{V_{max}*{\left[UTP\;or\;dUTP\right]}^{n}}{{K_{A}}^{n}+{\left[UTP\;or\;dUTP\right]}^{n}}$$

where $K_{d}={K_{A}}^{n}$

The *V_max_, n, K_A_*, and *K_d_* of UTP activity are 2.09 ± 0.09 U/mg, 2.52 ± 0.61, 0.30 ± 0.03 mM, 0.05 ± 0.04 mM, respectively. The *V_max_, n, K_A_*, and *K_d_* of dUTP activity are 0.71 ± 0.03 U/mg, 1.70 ± 0.19, 0.48 ± 0.04 mM, 0.28 ± 0.07 mM, respectively (Table S3).

The higher *V_max_* of UTP activity indicates that the reaction rate of CTPS using UTP is much higher than using dUTP. The lower K_d_, (measuring binding affinity) of UTP activity also indicates that CTPS prefers to bind to UTP rather than to dUTP. We also observed a positive cooperativity for both UTP and dUTP activities, with UTP showing a higher cooperativity. In general, dUTP can serve as a much slower but feasible substrate for CTPS activity.

We then proceeded to search for the structural basis of these catalytic differences. Models are aligned by the first 42 amino acids (Figure 3D). Compared with 4Pi-UTP, a shift from 4Pi dUTP to ATP/dATP binding pocket was observed, with the α, β and γ phosphate atoms moving 0.80, 0.84, and 0.82 Å, respectively. The hydrogen bond interaction between E154 and the 3′OH group of ribose or deoxyribose is conserved in both DON state and dDON state.

The absence of the 2′OH group on dUTP’s ribose ring appears to result in the loss of hydrogen bond interaction with D152. This leads to possible ligand position shifts that may affect enzymatic reaction, as well as weaker interactions between the ribose ring and CTPS for ligand recognition and stabilization.

Our map provides a structural basis for CTPS to catalyze dUTP as a substrate. These structural and conformational differences of CTPS between UTP state and dUTP state also explain their distinct kinetic behaviors.

### The allosteric regulation of CTPS activity induced by dGTP is weaker than that of GTP

**Figure 4:**
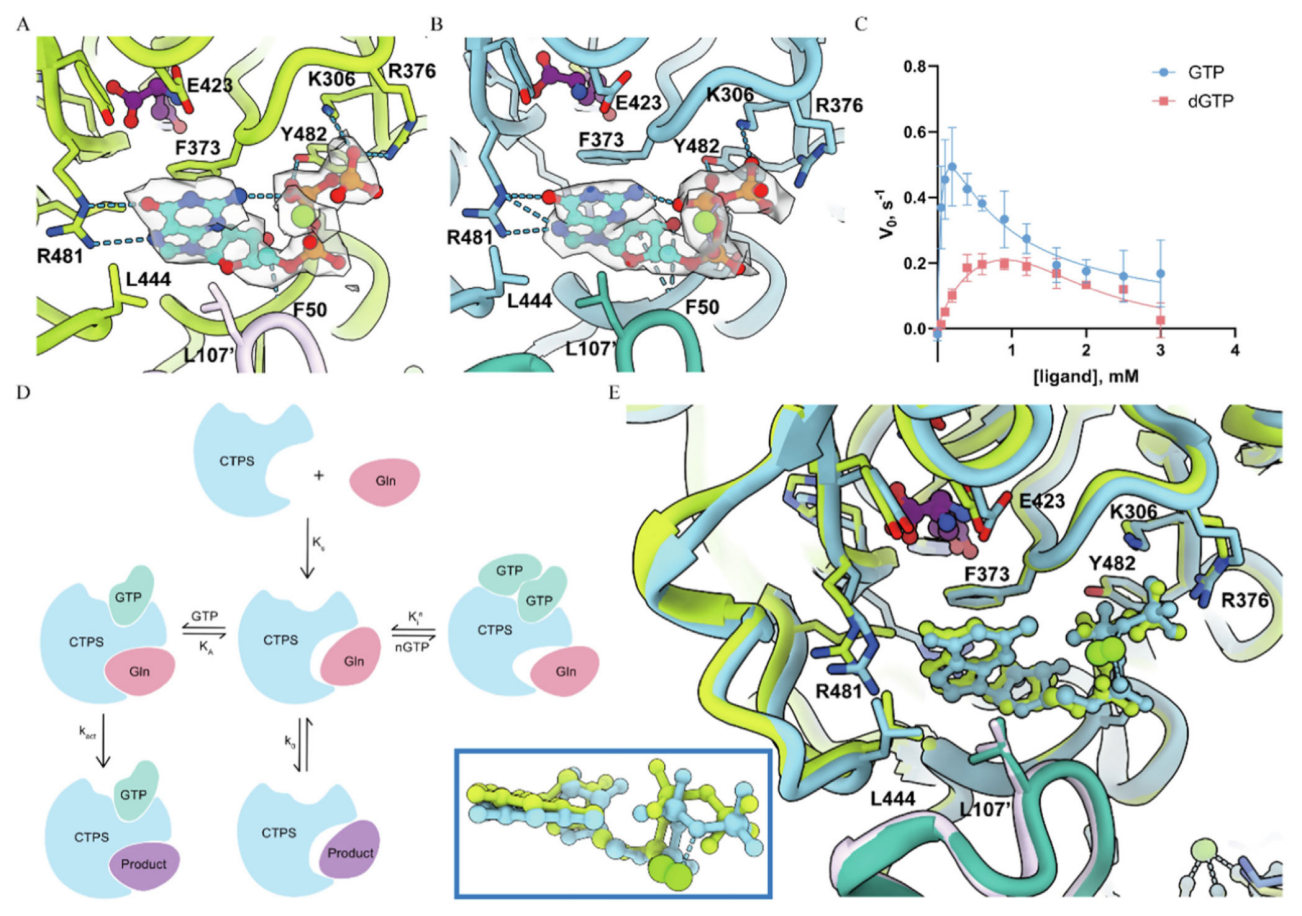
The structural, kinetic, and schematic characteristics of CTPS in GTP and dGTP binding modes. A, dGTP binding site in CTPS under dDON state (in this study). Mapping counter level: 7σ. B, GTP binding site in CTPS under DON state (PDB 7DPT). Mapping counter level: 7σ. C, Kinetic curves of CTPS with the allosteric regulator being either GTP or dGTP. The X-axis indicates ligand concentration. The Y-axis indicates the initial velocity of the enzymatic reaction. D, A schematic diagram of different regulatory modes of GTP on glutamine dependent CTP formation in CTPS, modified based on scheme by Bearne et al. This scheme is also applicable to the regulation of dCTP formation in CTPS by dGTP. E is CTPS; P is CTP; A is GTP or dGTP; S is Glutamine; K_A_ is the dissociation constant of the E·GTP complex; K_S_ is dissociation constant for glutamine; ${\text{K}}_{\text{i}}^{2}$ is the dissociation constant of the E·GTP^2^ complex; The number of GTP that associates with the enzyme in E·GTP^2^ complex is 2, corresponding to the parameter “n” in the kinetic model equation; k_act_, k_0_ and k_inh_ are the rates of CTP formation. E, Structural comparison between GTP binding pocket in DON state (blue) and dGTP binding pocket in dDON state (green). The blue box displays the detailed conformation differences between GTP and dGTP.

In the map generated by focus refinement, we observed a clear electron density of dGTP (Figure 4A). Similar to GTP in the DON state structure, dGTP is stabilized through multiple interactions between two different protomers (Figure 4B). This structural information supports the binding and regulatory role of dGTP in CTPS.

We then performed kinetic measurements of CTPS activity under GTP and dGTP regulation separately (Figure 4C). Considering the complex behavior exhibited by GTP and dGTP as allosteric regulators in CTPS activity, we optimized the biophysical model created by Bearne et al ^22,30^ and analyzed the kinetic data using this model. In the presence of saturating concentration of glutamine (10 mM), the kinetic model equation is as follows:

$$V_{0}=\frac{k_{0}+k_{act}*\frac{\left[GTP\;or\;dGTP\right]}{K_{A}}}{1+\frac{\left[GTP\;or\;dGTP\right]}{K_{A}}+\frac{{\left[GTP\;or\;dGTP\right]}^{n}}{K_{i}}}$$

The core idea of this model is that CTPS (as an enzyme, E) can form a complex with a GTP or dGTP, thereby forming an E·GTP complex or E·dGTP complex. In addition, CTPS can form complexes with multiple GTPs or dGTPs, resulting in the formation of an E·GTP^n^ complex or E·dGTP^n^ complex (Figure 4D).

According to our data, the activity of CTPS can be ignored without GTP/dGTP, indicating that *k*_0_ is close to 0. To simplify the fitting process of numerous parameters in this model, we reasonably assume that k_0_ is 0. Therefore, when *k_act_, K_A_, K_i_*, and *n* are 0.7741 ± 0.3879 s^−1^, 0.0511 6 ± 0.05114 mM, 0.1743 ± 0.05846 mM, and 1.95 5 ± 0.3757, respectively, GTP driven activity is most suitable for this model. When *k_act_, K_A_, K_i_*, and *n* are 0.3763 ± 0.1337 s^−1^, 0.5225 ± 0.3124 mM, 1.188 ± 0.165 mM, and 3.587 ± 0.8164, respectively, the dGTP driven activity is most suitable for the fitted best to the model (Table S4).

The higher *v*_0_ and *k_act_* observed in GTP driven activity suggest that GTP appears to coordinate CTPS activity more effectively than dGTP. In addition, lower *K_A_* and *K_i_* values associated with GTP driven activity indicate a greater likelihood of GTP binding to CTPS to form an E·GTP complex or E·GTP^n^ complex. It is also worth noting that the multisite inhibition model exhibits cooperativity, with dGTP driven activity (*n* ≈ 3 − 4) demonstrating stronger cooperativity compared to GTP-driven activity (*n* ≈ 2). These results are consistent with the observation, that is, a higher concentration of dGTP and a wider coverage range are required to achieve high enzyme activity (Figure 4B).

To elucidate the structural basis of the difference in CTPS regulation between GTP and dGTP, we compare the CTPS structures of DON state and dDON state. Structures are aligned by GAT domains, which also exhibit a similar conformation with an RMSD of 0.55 Å.

The main difference between GTP and dGTP binding modes lies in their hydrogen bond interactions with the backbone of F50. The absence of the 2′OH in dGTP’s ribose weakens its interaction with F50, which may be the reason why dGTP and GTP exhibit different conformations. This may further affect the interaction between R481 and the regulator, as well as the closed position of the wing (loop 440–448), thereby weakening the regulatory effect of dGTP on CTPS.

In summary, the interaction between dGTP and CTPS is not as strong as GTP, its activation of the reaction is weaker and the concentration of dGTP required to achieve its maximum activation is higher.

### Both CTP and dCTP can induce filament formation and inhibit the catalytic activity of CTPS

**Figure 5:**
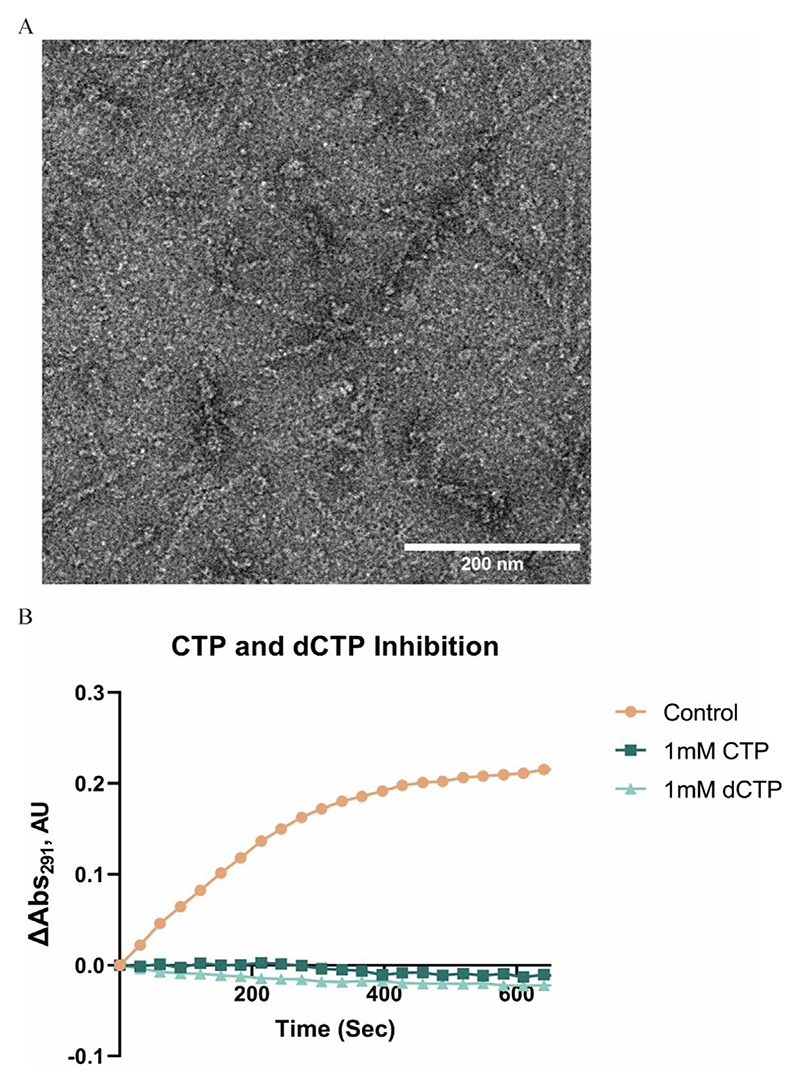
The effect of CTP and dCTP on CTPS. A, Negative staining electron microscopy image of 3.2 μM CTPS mixed with 1 mM dCTP and 4 mM MgCl_2_. B, The absorbance readings of CTPS activity under different conditions. The control condition is 12.5 mM Tris–HCl (pH 8.0), 75 mM NaCl, 10 mM MgCl_2_, 1 mM ATP, 1 mM UTP, 0.2 mM GTP, 10 mM Glutamine, and 2 μM CTPS. In addition, 1 mM CTP and 1 mM dCTP represent the absorbance readings of CTPS activity under the control condition with the addition of 1 mM CTP and 1 mM dCTP, respectively.

Carman’s group previously demonstrated that *URA7* in *Saccharomyces cerevisiae* can form tetramers under dCTP and CTP conditions.^67^ To further explore this phenomenon, we used negative staining electron microscopy to observe CTPS mixed with dCTP (Figure 5A). Under this condition, CTPS filaments form, indicating that dCTP can induce CTPS filamentation. We also performed a series of activity assays on CTPS to qualitatively validate the inhibitory effects of CTP and dCTP (Figure 5B). Under CTP and dCTP conditions, we observed no enzymatic activity. This indicates that both CTP and dCTP have strong inhibition effects on CTPS activity.

## Discussion

CTPS is the only enzyme that catalyzes the final step in de novo pyrimidine synthesis, producing CTP, which is essential to all biological lives. Previous studies have revealed the potential of CTPS to utilize deoxyribonucleotides. In 1999, Carman’s group reported that URA7 could utilize dUTP as a substrate to generate dCTP.^67^ Later, Endrizzi et al. reported that dATP and ATP were equally effective for *E.coli* CTPS.^29,44^

In this study, we have uncovered the structural basis of the dNTP dependent catalytic specificity of CTPS by analyzing the CTPS structure in the dDON state. Meanwhile, in order to systematically investigate the dNTP dependent catalytic mechanism of CTPS, we compare the kinetic differences among all nucleotide substrates and regulators. It is worth noting that this mechanism suggests an alternative pathway for the biological synthesis of dCTP.

Our research deepens our understanding of CTPS structure and validates previous work through orthogonal structural information. Using cryo-EM, we obtain maps with resolutions up to 2.73 Å. These high-resolution maps enable us to determine the precise binding modes of dNTPs, as compared to NTPs, and assess their interactions with CTPS. The structural differences support our kinetic analysis and indicate the catalytic mechanism of dNTP dependent CTPS activity.

**Figure 6:**
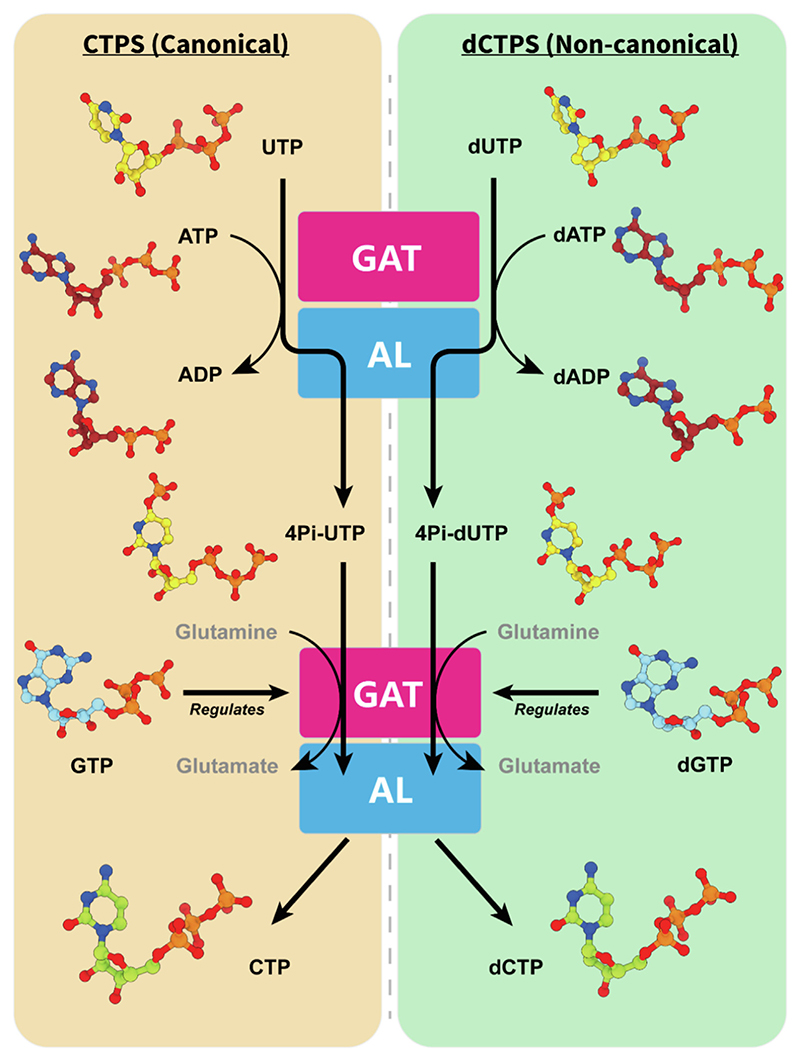
Catalytic diagrams of canonical CTPS and noncanonical dCTPS. As is well known, CTPS is the ratelimiting enzyme that catalyzes the final step of de novo biosynthesis of CTP, involving all four NTPs that assemble RNA. The same enzyme catalyzes the production of CTP and dCTP, highlighting its dual functions: canonical CTPS and noncanonical dCTPS. GAT represents the glutamine amide transferase (GAT) domain of CTPS/dCTPS; AL represents the ammonia ligase (AL) domain of CTPS/dCTPS.

In summary, our work contributes to the research on the dual functionality of nucleotide metabolic enzymes, specifically CTP/dCTP synthase (canonical CTPS and noncanonical dCTPS) (Figure 6). We have expanded upon previous studies by providing a structural explanation for the filamentation and catalysis of CTPS in the presence of dNTP substrates. This new insight into the structure of CTPS improves our understanding of the enzyme, particularly in regards to the binding of nucleotides. This knowledge may be useful in the development of potential CTPS substrates and regulators.

## Materials and Methods

### Protein expression and purification

A full-length *Drosophila melanogaster* CTPS sequence was constructed with a C-terminal 6XHis-tag and driven by T7 promoter. The plasmid was then transformed into Transetta (DE3) cells for expression. The cells were induced with 0.1 mM IPTG and incubated overnight at 16 ° C. Then, the cells were pelleted by centrifugation at 4,000 rpm for 15 min, and resuspended in cold lysis buffer (500 mM NaCl, 50 mM Tris–HCl (pH7.5), 20 mM imidazole, 1 mM PMSF, 7 μM leupeptin, 1.66 mM β-Me). The cell lysate was then centrifuged (18,000 rpm) at 7 °C for 1 h. Supernatant was collected and mixed thoroughly with equilibrated Ni-Agarose (Qiagen) for 1 h.

Subsequently, Ni-Agarose was washed with washing buffer (500 mM NaCl, 50 mM Tris–HCl (pH7.5), 64 mM imidazole, 5 mM β-Me). The protein was then eluted with elution buffer containing 500 mM NaCl, 50 mM Tris–HCl (pH7.5), and 266 mM imidazole. Hiload 16/600 Superdex 200 pg column and AKTA Pure (GE Healthcare) were used for further purification. Finally, CTPS was eluted with buffer containing 150 mM NaCl and 25 mM Tris–HCl (pH 8.0).

### Enzyme assays and kinetic data analysis

The conversion of UTP to CTP was detected by measuring the increase in absorbance at 291 nm on a SpectraMax i3 (Molecular Devices) spectrophotometer to determine UTP dependent CTPS activity. UTP and CTP have extinction coefficients of 182 and 1520 M^−1^ cm^−1^, respectively. Similarly, dUTP-dependent CTPS activity was determined by detecting the conversion of dUTP to dCTP through measuring the increase in absorbance at 291 nm. The extinction coefficients of dUTP and dCTP are 137 and 1801 M^−1^ cm^−1^, respectively. The standard reaction mixture for UTP-dependent CTPS activity contained 12.5 mM Tris–HCl (pH 8.0), 75 mM NaCl, 10 mM MgCl_2_, 1 mM ATP, 1 mM UTP, 0.2 mM GTP, 10 mM Glutamine, and 2 μM CTPS.

For dUTP dependent CTPS activity, the reaction mixture composition is the same, except that 1 mM UTP is substituted with 1 mM dUTP. When conducting kinetic analysis, the appropriate component of the standard reaction mixture is changed to investigate how different concentrations of a certain component impact the overall enzymatic activity. Enzyme concentration was determined by BCA assay. Kinetic data were analyzed using the GraphPad Prism 9.

### Negative staining electron microscopy

CTPS protein (3.175 μM) was dissolved in buffer containing 150 mM NaCl and 25 mM Tris–HCl (pH 8.0), 8 mM MgCl_2_, and 1 mM dCTP. After 30 min incubation at 37 °C, 5 μL samples were loaded onto hydrophilic carbon-coated grids (400mech, Zhongjingkeyi Technology Co., China). Excess protein was blotted off from the grids before dying the grids. The grids were washed twice with 3.5 μL 0.5% uranium formate and then dyed a third time with 3.5 μL 0.5% uranium formate, which stayed on the grids for about 40 sec before being blotted off. Imaging was performed on a 120 kV microscope (Talos L120C, ThermoFisher, USA) with an Eagle 4 K × 4 K CCD camera system (Ceta CMOS, ThermoFisher, USA). Images were acquired at 22,000×, 36,000×, and 57,000× magnification, respectively.

### Cryo-EM grid preparation and data collection

The Cryo-EM sample preparation conditions are the same as those for negative staining electron microscopy. Amorphous alloy film (No. M024-Au300-R12/13) was hydrophilized for 40 sec before applying the 2.7 μl sample solution. Then the grid was plunge-freezing by FEI Vitrobot (8 °C temperature, 3 sec blotting time, −1 blot force). Gatan K3 summit camera with 29,000X magnification in super-resolution mode on a 300 kV FEI Titan Krios electron microscope collected 4,497 movies. The defocus was set to 0.8–1.6 μm, and the raw pixel size was 0.41 Å. The total dose was 59.5 e^−^/Å^2^, divided into 50 frames at 2.5-sec exposure using SerialEM.

### Image processing

Cryo-EM data were processed by cryosparc V4.5.1.^68^ After patch motion correction and CTF estimation, particles were automatically picked from 4,287 selected images using references generated by manual picking and 2D classification. After data cleaning through 2D and 3D classification, 639,301 particles were selected from 3,709,479 for homogeneous refinement. D2 symmetry was used for the final round of 3D classification and 3D reconstruction. The first round of D2 symmetric homogeneous refinement resulted in a map of 2.93 Å.

After CTF Refinement and Bayesian Polishing, the resolution of the map was improved to 2.79 Å. The helical rise and twist were determined by fitting tetramers into this map. To further improve the resolution of the map, density subtraction was performed to eliminate the influence of the surrounding density from the central tetramer. A round of 2D classification was performed to validate the quality of density subtraction. Finally, a 2.73 Å map was obtained for CTPS tetramer.

To improve the quality of individual protomers, focus refinement was conducted. Initially, particles of the 2.79 Å map obtained through homogeneous refinement were expanded with D2 symmetry. Then one round focus classification was performed to exclude particles with poor quality on the edge of protomers. In the end, 832,381 particles were selected for the local refinement, resulting a map with better local quality at the same overall resolution.

### Model building and refinement

7DPT was used as the initial model for model building. The manual fitting of models was completed using COOT.^69^ Then, the coordinates were verified and tuned by Phenix.^70^

## Supplementary Material

Supplementary material to this article can be found online at https://doi.org/10.1016/j.jmb.2024.168750.

Supplementary file

## Data Availability

Data will be made available on request.
