# Supplementary material for: Structural Basis of Bifunctional CTP/dCTP Synthase
Source: J Mol Biol. Author manuscript; Available in PMC 2026 Apr 14. (PMC7619001; doi:10.1016/j.jmb.2024.168750)
Supplement: Supplementary file [file EMS213046-supplement-Supplementary_file.docx]

**Extended Data**

**
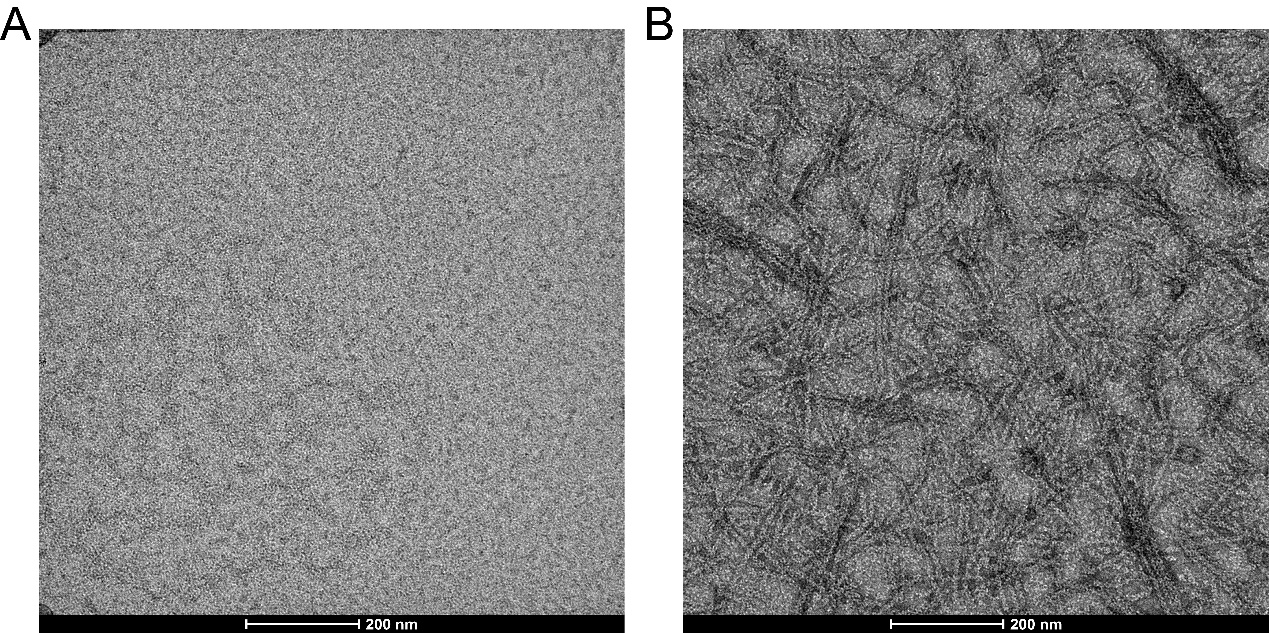
**

**Supplementary Fig. S1. Negative staining image of *Drosophila* CTPS.** A, Negative staining electron microscopy image of 2 μM *Drosophila* CTPS mixed with 10 mM Gln and 10 mM MgCl_2_. B, Negative staining electron microscopy image of 2 μM *Drosophila* CTPS mixed with 10 mM Gln, 1 mM ATP, 1 mM UTP and 10 mM MgCl_2_. Scale bars, 200 nm.


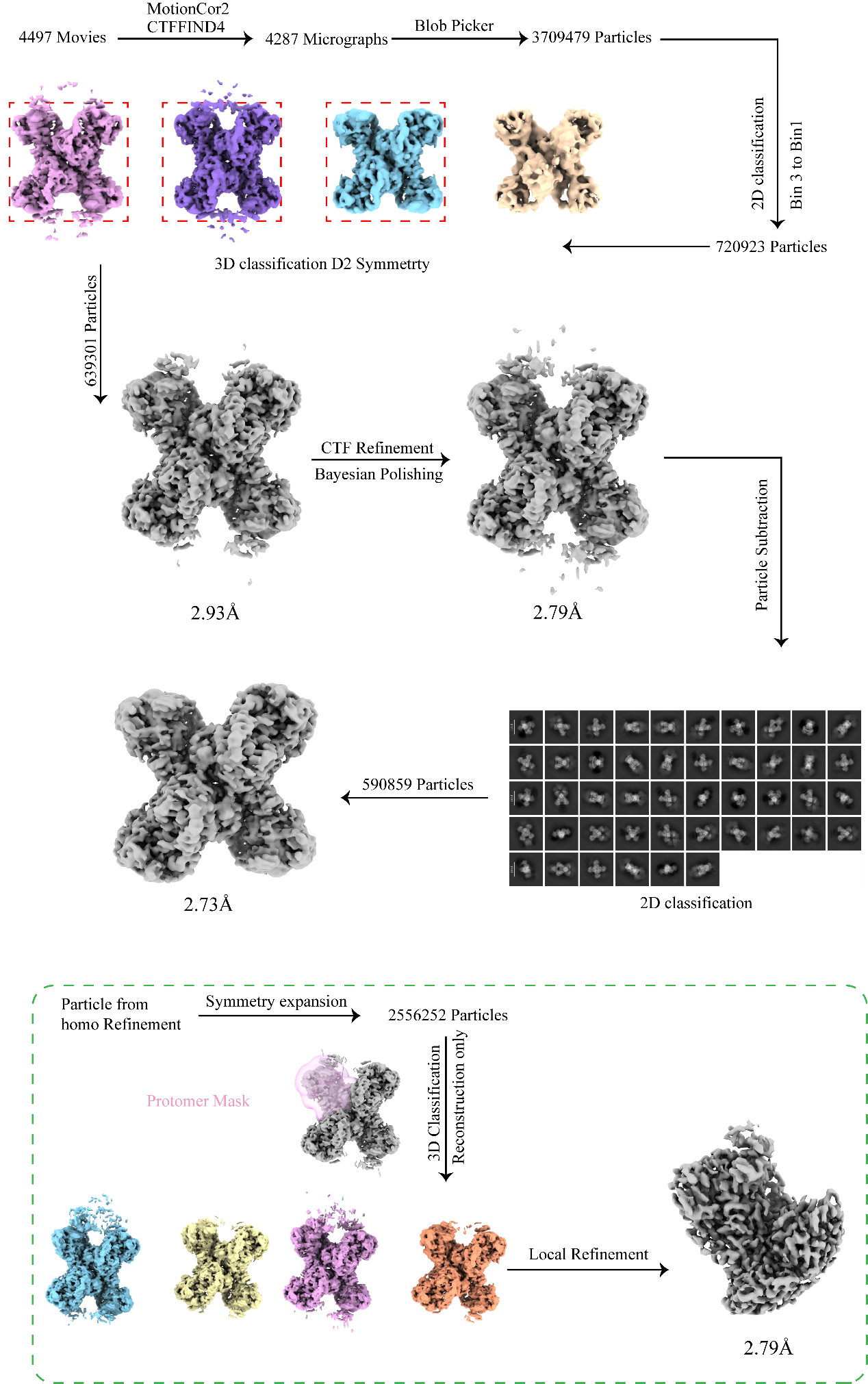


**Supplementary Fig. S2. The workflow of data processing.**

**
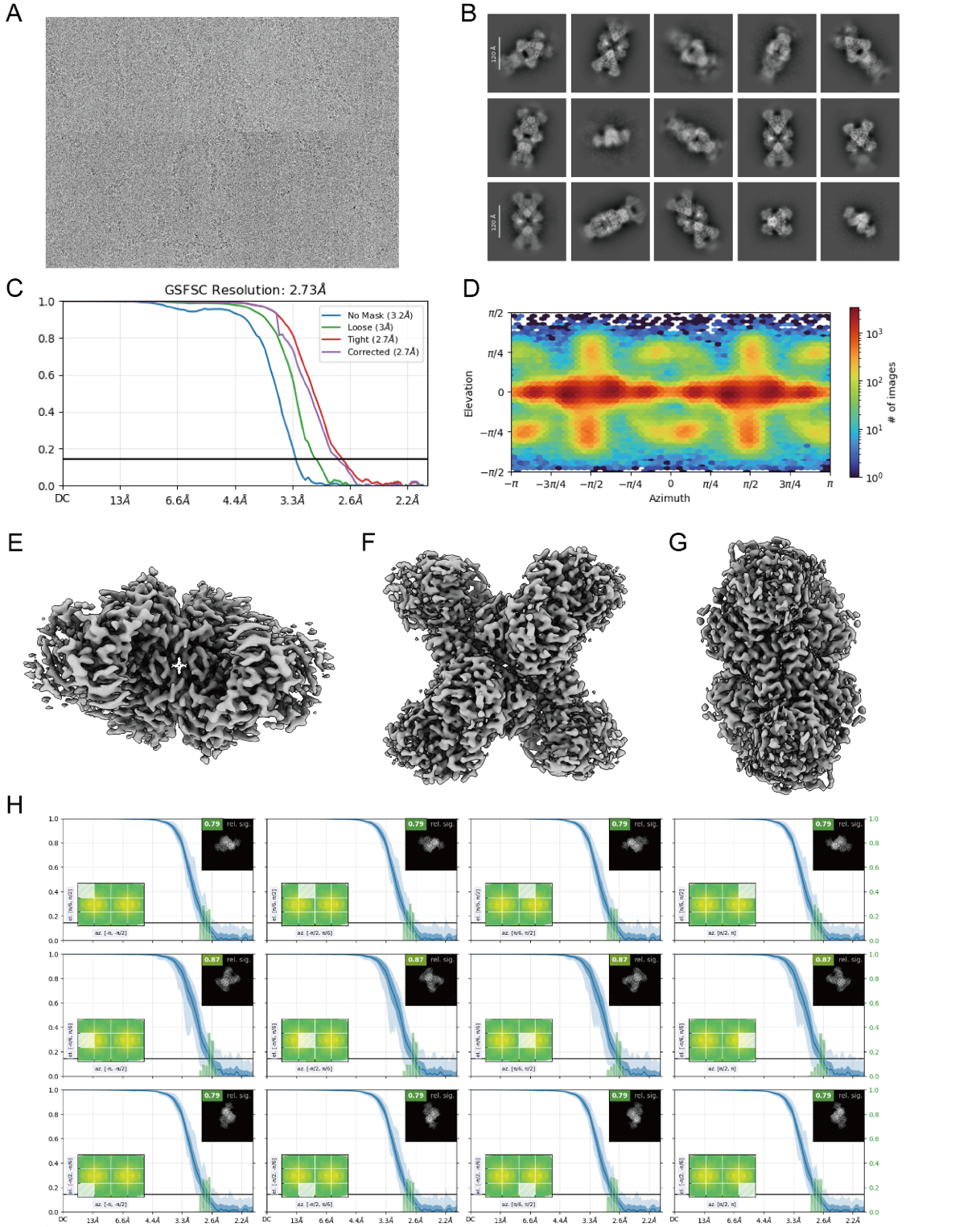
**

**Supplementary Fig. S3. Statistics of the final density maps of tetramers.** A, A representative image of raw data. B, Representative 2D classes. C, Gold standard FSC curve. D, Viewing direction distribution. E-G, Top view, front view, and side view of the map at level 0.1. H, The average relative signal quantity within Azimuth-Elevation viewing regions.


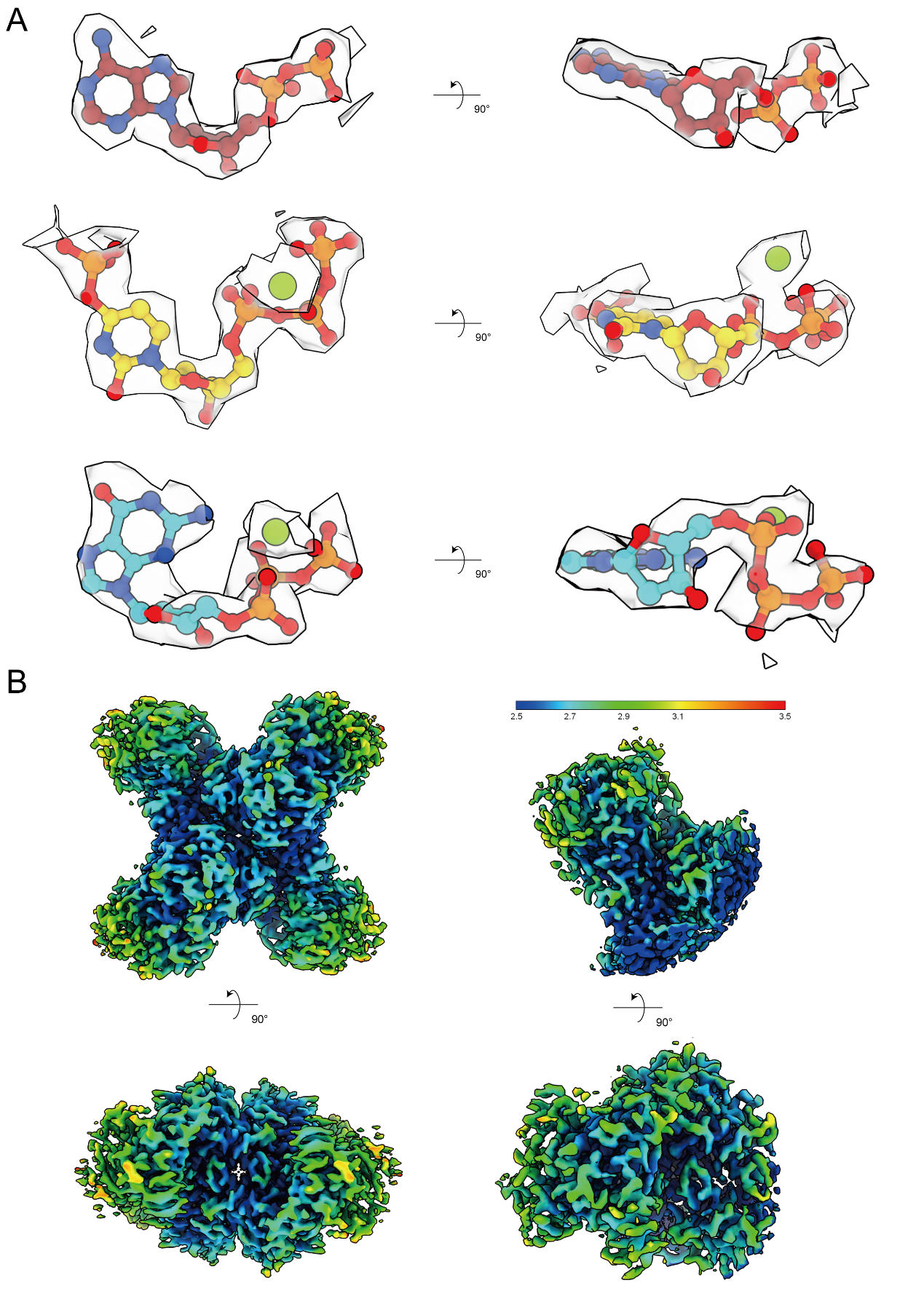


**Supplementary Fig. S4. Ligand models used for density display and local resolution estimation.** A, Models and densities of dADP, 4Pi-dUTP, and dGTP from different perspectives. B. Local resolution estimation of two final maps.


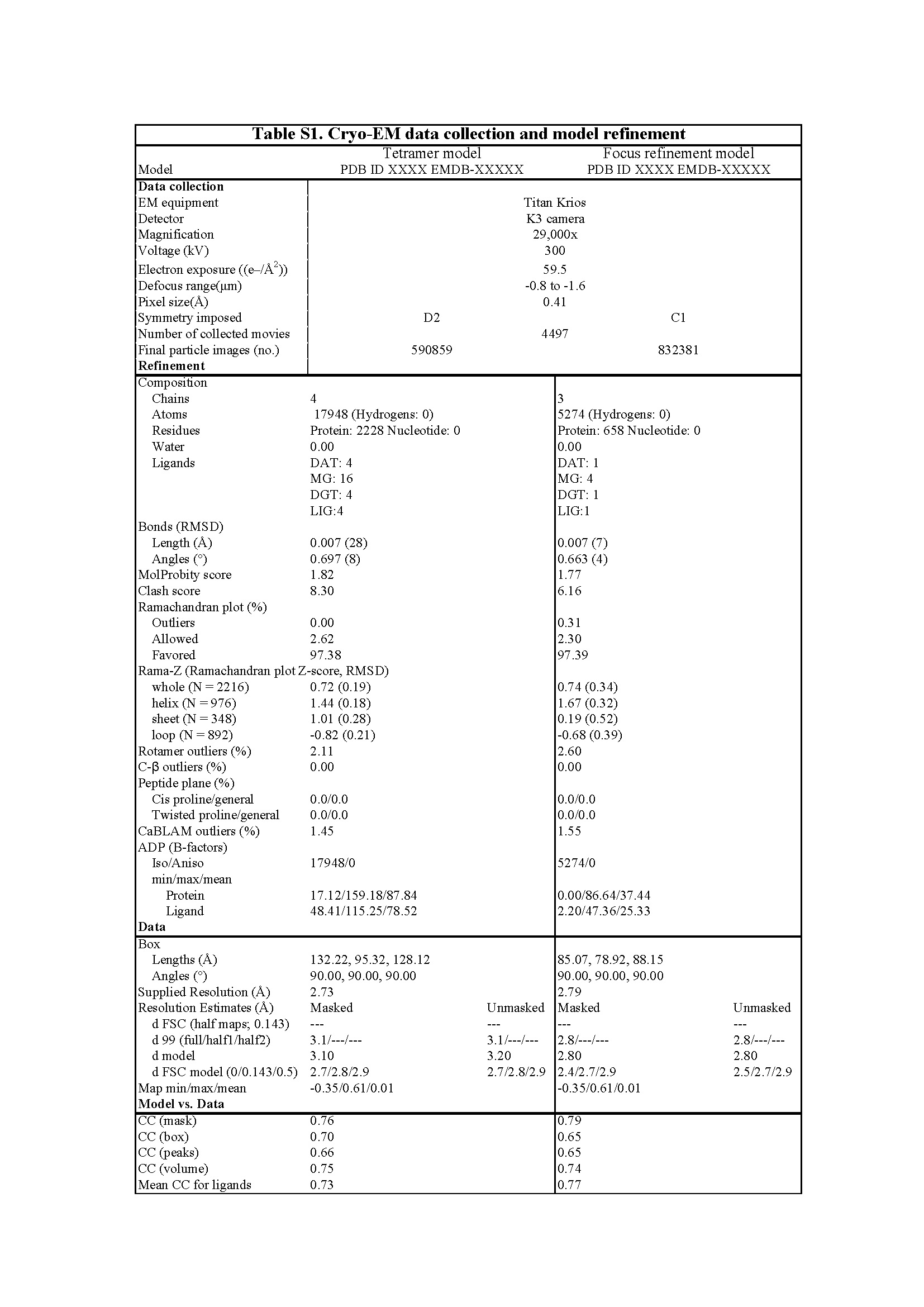


| Table S2. Comparison between ATP- and dATP-driven activities of CTPS^[[1]](#footnote-1)^ | | |
| --- | --- | --- |
|  | ATP-driven activity | dATP-driven activity |
| V_max_ | 1.87 ± 0.06 U/mg | 1.67 ± 0.10 U/mg |
| n | 1.67 ± 0.25 | 1.33 ± 0.26 |
| K_A_ | 0.19 ± 0.02 mM | 0.25 ± 0.04 mM |
| K_d_ | 0.06 ± 0.03 mM | 0.16 ± 0.08 mM |

| Table S3. Comparison between UTP- and dUTP-dependent activities of CTPS^[[2]](#footnote-2)^ | | |
| --- | --- | --- |
|  | UTP-dependent activity | dUTP-dependent activity |
| V_max_ | 2.09 ± 0.09 U/mg | 0.71 ± 0.03 U/mg |
| n | 2.52 ± 0.61 | 1.70 ± 0.19 |
| K_A_ | 0.30 ± 0.03 mM | 0.48 ± 0.04 mM |
| K_d_ | 0.05 ± 0.04 mM | 0.28 ± 0.07 mM |

| Table S4. Comparison between GTP- and dGTP-driven activities of CTPS | | |
| --- | --- | --- |
|  | GTP-driven activity | dGTP-driven activity |
| k_act_ | 0.7741 ± 0.3879 s-1 | 0.3763 ± 0.1337 s-1 |
| K_A_ | 0.05116 ± 0.05114 mM | 0.5225 ± 0.3124 mM |
| K_i_ | 0.1743 ± 0.05846 mM | 1.188 ± 0.165 mM |
| n | 1.955 ± 0.3757 | 3.587 ± 0.8164 |

1. Activity is measured with a fixed concentration of UTP and GTP at 1mM and 0.2mM, respectively. [↑](#footnote-ref-1)
2. Activity is measured with a fixed concentration of ATP and GTP at 1mM and 0.2mM, respectively. [↑](#footnote-ref-2)
